# A catchment‐scale perspective of plastic pollution

**DOI:** 10.1111/gcb.14572

**Published:** 2019-02-20

**Authors:** Fredric M. Windsor, Isabelle Durance, Alice A. Horton, Richard C. Thompson, Charles R. Tyler, Steve J. Ormerod

**Affiliations:** ^1^ School of Biosciences Water Research Institute, Cardiff University Cardiff UK; ^2^ Biosciences University of Exeter Exeter UK; ^3^ Centre for Ecology and Hydrology Wallingford UK; ^4^ Faculty of Science & Engineering Plymouth University Plymouth UK

**Keywords:** ecological risk, ecotoxicology, macroplastic, microplastic, pollution, river basin

## Abstract

Plastic pollution is distributed across the globe, but compared with marine environments, there is only rudimentary understanding of the distribution and effects of plastics in other ecosystems. Here, we review the transport and effects of plastics across terrestrial, freshwater and marine environments. We focus on hydrological catchments as well‐defined landscape units that provide an integrating scale at which plastic pollution can be investigated and managed. Diverse processes are responsible for the observed ubiquity of plastic pollution, but sources, fluxes and sinks in river catchments are poorly quantified. Early indications are that rivers are hotspots of plastic pollution, supporting some of the highest recorded concentrations. River systems are also likely pivotal conduits for plastic transport among the terrestrial, floodplain, riparian, benthic and transitional ecosystems with which they connect. Although ecological effects of micro‐ and nanoplastics might arise through a variety of physical and chemical mechanisms, consensus and understanding of their nature, severity and scale are restricted. Furthermore, while individual‐level effects are often graphically represented in public media, knowledge of the extent and severity of the impacts of plastic at population, community and ecosystem levels is limited. Given the potential social, ecological and economic consequences, we call for more comprehensive investigations of plastic pollution in ecosystems to guide effective management action and risk assessment. This is reliant on (a) expanding research to quantify sources, sinks, fluxes and fates of plastics in catchments and transitional waters both independently as a major transport routes to marine ecosystems, (b) improving environmentally relevant dose–response relationships for different organisms and effect pathways, (c) scaling up from studies on individual organisms to populations and ecosystems, where individual effects are shown to cause harm and; (d) improving biomonitoring through developing ecologically relevant metrics based on contemporary plastic research.

## Graphical abstract

Most research into plastic pollution has focused on marine ecosystems, but these are only the endpoint for plastics of all forms that stem from terrestrial and freshwater environments, particularly rivers. Here, we review the occurrence and transfer of plastic pollution across multiple ecosystems, and advocate the investigation of plastic sources, fluxes and fates in hydrological catchments as well‐defined landscape units that provide an integrating scale for understanding and managing ecological effects.

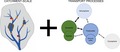

## 1 INTRODUCTION

Plastic waste production across the globe has reached approximately 6,300 million metric tons (MT), most (79%) of which has been disposed of to landfills and more widely into the surrounding environment (Geyer, Jambeck, & Law, 2017). The annual flow of plastic pollution to the world's oceans is estimated to be 4.8–12.7 MT, a large proportion of which comes from sources on land and is transported by rivers or wind (Jambeck et al., 2015). Plastic pollution is comprised of a variety of different organic polymers (e.g. polyethylene terephthalate, high‐density polyethylene, polyvinyl chloride, polyethylene, polypropylene and polystyrene) and is invariably categorized on size distribution. The size classification of plastic is variable across studies, yet here we identify: nano‐ (<100 nm), micro‐ (0.0001–5 mm), meso‐ (5–25 mm) and macroparticles (>25 mm). Once in situ within ecosystems, degradation and fragmentation processes make the identification and removal of these plastic particles difficult, particularly the smaller size fractions. Problems in managing plastic pollution, however, begin even earlier in their life cycle. Indeed, recent reviews and theoretical models have indicated a large number of potential sources, fluxes and sinks of plastics across the wider environment (Alimi, Farner Budarz, Hernandez, & Tufenkji, 2018; Browne et al., 2011; de Souza Machado, Kloas, Zarfl, Hempel, & Rillig, 2018; Horton, Svendsen, Williams, Spurgeon, & Lahive, 2017; Wagner et al., 2014). While crude estimates of environmental plastic fluxes have been attempted, a more detailed understanding of the sources, fluxes and effects of these anthropogenic pollutants in time and space, and a more comprehensive quantification of their fate, is now required urgently to determine the risks to people and ecosystems across the globe (de Souza Machado, Kloas et al., 2018; Horton & Dixon, 2017; Nizzetto, Bussi, Futter, Butterfield, & Whitehead, 2016).

Large production volumes, long‐term environmental persistence and potential ecological effects are now increasing attention on plastic pollution (Thompson, Swan, Moore, & vom Saal, 2009). The variety of plastic sizes (microns to metres) and characteristics (e.g. shape, physical and chemical properties) make this group of pollutants particularly diverse (Rochman, 2015). In turn, the diversity and ubiquity of plastic particles within natural systems means that there is a wide variety of ways organisms can interact with, become entangled in or ingest plastic particles (e.g. Cole et al., 2013; Foekema et al., 2013; Lusher, McHugh, & Thompson, 2013, Lusher, Hernandez‐Milian et al., 2015; Hall, Berry, Rintoul, & Hoogenboom, 2015). Although existing information indicates the potential for effects across biological communities and human populations (Halden, 2010), understanding of the effects of plastic pollution on people and ecosystems remains constrained. Furthermore, despite widely identified interactions between organisms and plastics, a comprehensive mechanistic understanding of effect pathways remains limited, with a few notable exceptions (e.g. ingestion and energy reserve depletion: Wright, Rowe, Thompson, & Galloway, 2013a). Existing dose–response relationships for effect pathways are not only restricted but also often limited across taxa or to unrealistic concentrations and plastic characteristics (Phuong et al., 2016). Emerging reviews have started to collate real or predicted no effect concentrations for several microplastic types and size categories, while also incorporating a range of aquatic organisms, but their scope is inevitably limited by the volume of available research (Burns & Boxall, 2018; Everaert et al., 2018).

In this review, we evaluate critically the existing evidence on the fluxes and effects of plastic pollution from a catchment‐scale perspective. We focus particularly on freshwater ecosystems as highly connected networks through which plastics are transported from sources in terrestrial environments to marine ecosystems. We aim to: (a) synthesize existing knowledge regarding the fluxes and effects of plastic pollution across hydrological catchments; (b) highlight emerging areas that require further research; and (c) identify improvements to aid the development and integration of catchment‐scale research that should ultimately inform management strategies.

## 2 FLUXES OF PLASTICS THROUGH HYDROLOGICAL CATCHMENTS

**Figure 1:**
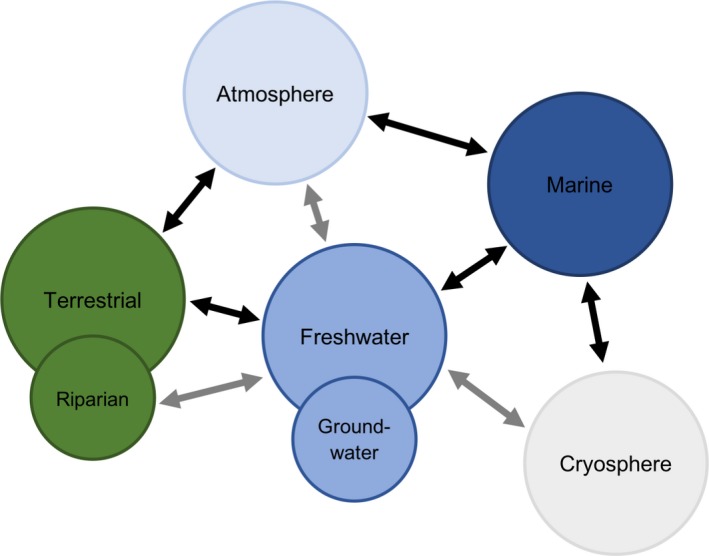
Conceptual diagram of plastic fluxes across the compartments of hydrological catchments. Specific pathways, indicated by black arrows, are further discussed within the main body of text. Grey arrows represent theoretical fluxes that have yet to be investigated in detail (see Underrepresented ecosystems)

Hydrologically defined river catchments are important units in which to consider the sources, fluxes and fates of plastic pollution (Figure 1). This is because the transport of plastics often follows hydrological pathways that are determined clearly by topography, surface morphology and drainage patterns from a wide range of land use types (Bracken et al., 2013).

Once released into the environment, plastics reach across all ecosystems and ecotypes across the globe (Geyer et al., 2017). Plastic particles are widespread, even in areas considered to have little to no human influence, such as the deep sea, Arctic sea ice and remote uninhabited islands (Lavers & Bond, 2017; Peeken et al., 2018; Peng et al., 2018; Van Cauwenberghe, Vanreusel, Mees, & Janssen, 2013). Along their movement from source to sink, plastics interact with the physical, chemical and biological environment in ways that depend on the characteristics of the plastic (size, shape, polymer type, etc.) so that it is not practical to consider “plastics” as a singular form of pollution. Nevertheless, for this discussion, we highlight existing theoretical and empirical evaluations of the flux and effects of a broad group of “plastics” (defined above) across ecosystems.

The movement of plastic among the compartments of river catchments is analogous to other catchment‐scale processes involving fluxes, transformations and storage (Horton & Dixon, 2017). It has been suggested theoretically that microplastic particles behave in a similar manner to other particulate matter with similar characteristics (e.g. density, size and shape), such that movement of these particles resembles the fluxes of others (e.g. sediment/soil particles, fine and coarse organic matter (Nizzetto, Bussi et al., 2016). In reality, however, it is likely that the unique diversity of shape, density, size or surface complexity of plastic particles, limits the accuracy and utility of existing models to predict plastic movement across and within ecosystems. Furthermore, the behaviour of larger particles of plastic (meso to macro) within ecosystems remains poorly understood. The processes responsible for transporting these larger particles are likely similar to those transporting microplastics, yet operate at larger scales, involve more energy and occur more sporadically. As a result of these complications, there remains insufficient data to accurately parameterize and validate empirical transport models for plastic pollution.

While the movement of plastic between atmospheric, terrestrial and freshwater systems appears to multidirectional, marine systems are generally perceived to act as sinks for plastics, with limited outflux (Browne et al., 2011). However, a significant amount of plastic is transported through river catchments (Lebreton et al., 2017). While this is likely to be the main source of marine plastics (Nizzetto et al., Nizzetto, Bussi et al., 2016), little is known about the residence time of plastics in streams, rivers and lakes, which could act as plastic “traps” that then increase organism exposure. Quantification of all the pathways from land to sea remains limited (but see Clark et al., 2016; Galloway, Cole, & Lewis, 2017) yet is key to supporting the estimation of ecological risk across systems.

The characteristics of hydrological catchments have important implications for the flux of plastic pollution across the landscape. Features such as topography, hydrology and land use are likely to be responsible for altering the mass balance of plastics within catchments – influencing both the diversity and volumes of plastic emitted from sources, the nature and magnitude of transport processes as well as the likelihood of temporary storage across ecosystems. Limited information exists at the catchment‐scale, however, and too few studies have quantified plastic movements at an appropriate scale. Here, however, we present findings from existing studies investigating plastic pollution across atmospheric, terrestrial, freshwater and marine systems to provide a generic basis for understanding catchment‐scale plastic transport.

### 2.1 Terrestrial systems

Several sources of plastic pollution are associated with human activities across the terrestrial environments present within hydrological catchments (de Souza Machado, Kloas et al., 2018; Hurley & Nizzetto, 2018) such that plastic pollution reflects a patchwork of point and diffuse sources in which both rural and urban soils are considered to be contaminated by plastic particles (Nizzetto, Futter, & Langaas, 2016). Intensive agricultural practices distribute plastics across rural regions through the degradation of machinery, diffuse littering, application of sewage sludge as a soil conditioner (Zubris & Richards, 2005) and plastic mulching (Steinmetz et al., 2016). The redistribution of sewage sludge is particularly interesting, transporting plastics of urban origin across some rural landscapes (Horton, Svendsen et al., 2017; Zubris & Richards, 2005). The flux of plastics from this activity is potentially important considering that 80%–99% of plastics entering sewage treatment are stored in sludge (Carr, Liu, & Tesoro, 2016; Talvitie, Mikola, Setälä, Heinonen, & Koistinen, 2017), and a large amount of MPs (4,196–15,385 MP/kg dry mass) remain post‐treatment of biosolids (Mahon et al., 2017). Within Europe, Nizzetto, Futter et al. (2016) estimated that 125–180 t of microplastics per million inhabitants are added to agricultural soils as a result of sewage sludge application. Urban land use and associated activities also provide several different sources of plastic pollution (Ballent, Corcoran, Madden, Helm, & Longstaffe, 2016; Nizzetto, Futter et al., 2016). In particular, loss during waste disposal, industrial spillage and release from landfills provide significant inputs of plastic (Lechner & Ramler, 2015; Sadri & Thompson, 2014). The large production of plastics in terrestrial systems, limited land area and range of distribution processes may result in a greater environmental concentration within these ecosystems, compared to marine environments (Horton et al., Horton, Svendsen et al., 2017).

The flux and storage of plastic within terrestrial systems have been catalogued theoretically, but there are few field data. Once in terrestrial ecosystems, plastics accumulated in surface soils and can be ingested by soil‐dwelling organisms (Rillig, 2012; Rillig, Ingraffia, & Souza Machado, 2017). Empirical data indicate that plastics are incorporated into earthworm casts (Huerta Lwanga et al., 2017), and also that polyethylene microbeads (0.71–2.8 mm) reach down into the subsurface through earthworm burrows (Rillig, Ziersch, & Hempel, 2017). The concentration of plastic in soils varies; river floodplains across Switzerland revealed relatively low concentrations of microplastics (0–55.5 mg/kg, Scheurer & Bigalke, 2018), but more heavily contaminated industrial soils (300–67,500 mg/kg) have been observed from samples collected in Australia (Fuller & Gautam, 2016). The lightweight nature of plastic material means that, in terrestrial systems, particles are more easily transported by wind and weather events (Zylstra, 2013), diffusing their distribution across catchments.

Plastics stored in terrestrial systems may subsequently be remobilized and transported within or across catchments (Dris, Gasperi et al., 2015; Duis & Coors, 2016; Wagner et al., 2014). Although empirical assessments are absent from the literature, soil erosion during heavy rainfall is likely to increase the flux of plastic particles from soils to river systems (Bläsing & Amelung, 2018). Landfill sites in low‐lying areas prone to flooding present a significant additional source of plastics into freshwater ecosystems (Brand et al. 2018). In some cases, as during flood events, plastics may even return to land; however, the flow of plastics out of terrestrial systems appears dominant and drives the global plastic cycle (see de Souza Machado, Kloas et al., 2018).

### 2.2 Atmospheric systems

Plastic, as a result of its lightweight characteristics, can be suspended and transported within the atmosphere at both the catchment and regional scale (Dris, Gasperi, Saad, Mirande, & Tassin, 2016; Prata, 2018). Plastics enter the atmospheric system through a variety of pathways across catchments, including combustion of waste plastic, wind erosion of various media, urban dust (including tyre wear particles, paint particles and synthetic fibres) (Lee et al., 2016; Unice, Kreider, & Panko, 2012) and diffuse litter (Dris et al., 2016). The majority of plastic in the atmosphere falls into the micro‐ and nano‐size classes; nevertheless, larger particles may be suspended in the atmosphere if they have certain characteristics (e.g. disposable plastic bags and balloons). Significant concentrations of plastic are observed within the lower atmosphere (0.3–1.5 MPs/m^3^), yet compared to indoor air, these values are relatively low (1–60 MPs/m^3^) (Dris et al., 2017). Polyurethane, polypropylene and polystyrene microplastic particles were identified in atmospheric fallout, at concentrations between 175 and 313 MP m^−2^ day^−1^ in Dongguan city (Cai et al., 2017). Similar concentrations of microplastic were also observed using passive samplers in Paris, 2–355 MPs m^−2^ day^−1 ^(Dris et al., 2016). The fallout of these particles is, in turn, responsible for the accumulation of particles in “street dust”. For example, “street dust” collected from sites across Tehran exhibited 2,933–20,166 MP kg^−1^ (Dehghani, Moore, & Akhbarizadeh, 2017). The atmosphere, therefore, appears to store and transport plastic, and while there is limited evidence of long‐range atmospheric flows of plastic, microplastic pollution occurs in remote environments such as alpine lakes (Free et al., 2014). The storage and transportation of plastics in the atmosphere are likely temporally variable, influenced by the prevailing meteorological conditions at different timescales. Thus, it is unlikely that the atmosphere provides a long‐term store of plastics, instead acting as a temporary store, as well as a potential short‐ and long‐distance transport pathway.

### 2.3 Freshwater systems

Freshwater ecosystems include a diverse array of running, standing, surface and underground waterbodies. Running waters act as conduits connecting terrestrial, freshwater, transitional and marine systems, providing an important long‐range transport pathway as well as storage opportunities in some benthic, floodplain or riparian habitats (Horton & Dixon, 2017). Standing waters, including lakes and ponds, may also accumulate and store plastic (Vaughan, Turner, & Rose, 2017). The role of freshwaters in the transport of plastics across catchments is likely to be highly dependent on the characteristics of waterbodies, yet systematic quantification is limited.

The sources of plastic entering freshwater ecosystems are varied and spatially heterogeneous, ranging from diffuse inputs stemming from run‐off to point sources such as Wastewater Treatment Works (WwTWs) and Combined Sewer Overflows (CSOs) (Horton, Svendsen et al., 2017). Domestic sewage collects a variety of plastic types, including synthetic wet wipes, microbeads (Duis & Coors, 2016) and polymer fibres from the laundering of synthetic textiles (Napper & Thompson, 2016). WwTWs effectively remove the vast majority of both large and small plastics from raw influent (95%–99%), yet these point sources remain an important contributor of smaller microplastic particles directly into freshwater ecosystems (Murphy, Ewins, Carbonnier, & Quinn, 2016; Talvitie et al., 2017). These contributions from treated effluent, however, are spatially variable in response to variable removal efficiencies across WwTWs (Siegfried, Koelmans, Besseling, & Kroeze, 2017). Microplastics removed during treatment are also not completely disconnected from entering the environment, with the retention of plastics in sludge (Mahon et al., 2017) and the potential for subsequent reapplication across catchments. Further sources of micro‐ and macroplastics identified within existing literature include, diffuse urban pollution, storm water drains (Horton, Walton, Spurgeon, Lahive, & Svendsen, 2017), combined sewage overflows and litter (Horton, Svendsen et al., 2017). The combined effects of urban pollution sources have been shown to generate enhanced concentrations of plastics within freshwater systems, for example, the highly populated Lake Erie maintains far greater concentrations of microplastic particles (43,000 MP/km^2^) in comparison to lakes in proximity to less populated regions, for example, Lake Huron (6,541 MP/km^2^) and Lake Superior (12,645 MP/km^‐2^) (Eriksen et al., 2013). As a result of the ubiquity of point and diffuse sources of plastic pollution within freshwaters, it is not surprising that plastic has been widely identified within a range of freshwater habitats (Free et al., 2014; Horton, Walton et al., 2017). Data from freshwater systems, thus far, indicate that these systems are important hotspots of plastic pollution, holding some of the highest concentrations of (micro)plastics recorded in either water and sediments across the globe (Hurley, Woodward, & Rothwell, 2018; Mani, Hauk, Walter, & Burkhardt‐Holm, 2015).

River systems act as conduits, connecting terrestrial, riparian, floodplain and transitional ecosystems within their catchments. Theoretical and modelling assessments support the notions of particle transfer across habitats, but also demonstrate significant storage under certain conditions (see Nizzetto, Bussi et al., 2016). The retention and transport of plastics are a product of particle characteristics (density and dimensions) and environmental characteristics (flow regime) (Nizzetto, Bussi et al., 2016). Within river systems, plastics may pool in benthic sediments (Castañeda, Avlijas, Simard, & Ricciardi, 2014) or be transferred along an altitudinal gradient towards marine ecosystems (Lebreton et al., 2017; Mani et al., 2015). This transport may occur throughout the water column, with significant transport observed both on the surface (Dris, Imhof et al., 2015; Lechner et al., 2014) and subsurface (Morritt, Stefanoudis, Pearce, Crimmen, & Clark, 2014) of river systems.

The interaction between storage and flux processes is highlighted in a recent study by Hurley et al. (2018), which indicates the significant mobilization and removal of sedimentary microplastics in response to high flow events. In this example, 0.85 ± 0.27 tonnes of plastic was removed from a single catchment during an individual flood event (Hurley et al., 2018). Similar flood events may also be responsible for distributing plastics onto floodplains. The net or total flux of plastics from terrestrial sources, through hydrological networks to marine systems, however, remains poorly understood. It is, however, estimated that global river networks are responsible for transferring 1.15–2.41 MT of plastic pollution to marine environments (Lebreton et al., 2017). This estimate, however, is based solely on surface transport and does not account for suspended and bedload transport. As a result, the mass of plastic transported through river systems are likely to be underestimated, with the combination of surface and subsurface transport more likely accounting for a greater proportion of the total 4.8–12.7 MT estimated entering marine environments per year (Jambeck et al., 2015).

### 2.4 Marine systems

Oceans are often considered the endpoint of plastic fluxes from hydrological catchments (Horton & Dixon, 2017). As highlighted previously, it is estimated that fluxes of plastics from rivers provide a major input of macro‐ and microplastics into marine environments across the globe (Lebreton et al., 2017; UNEP, 2016). With 50% of the global population residing within 31 km of the coast (Small & Cohen, 2004), direct inputs of plastics are also likely to be significant. Finally, industrial activity, such as commercial fishing, contributes to the total plastic burden within marine ecosystems (Lusher, Tirelli, Tirelli, O'Connor, & Officer, 2015). In most cases, these activities release macroplastics, such as netting and plastic sheeting, which then degrades to form microplastic particles when exposed to physical, chemical or biological processes (e.g. Davidson, 2012). The potential variety of plastic sources generates a widespread distribution of plastics in the marine environment, yet heterogeneity exists with accumulation zones and plastic hotspots (Lusher, 2015). Plastic transport processes are widespread and heterogeneous within the marine environment (Browne et al., 2011). Ocean and wind circulation currents, ranging from small‐scale vertical mixing to large‐scale oceanic gyres, appear responsible for the observed patchiness of plastic distribution within marine systems (Kukulka, Proskurowski, Morét‐Ferguson, Meyer, & Law, 2012; van Sebille et al., 2015). In coastal regions, local hotspots may also be generated by the influx of plastics from river systems (Frias, Otero, & Sobral, 2014).

Although not commonly appreciated, plastics are also transported out of marine and coastal ecosystems to terrestrial and atmospheric environments through wind and wave action (e.g. storm surges) (Hoffmann & Reicherter, 2014; Horton et al., Horton, Svendsen et al., 2017). These transport pathways redeposit plastic to coastal/terrestrial systems. For example, a large proportion of plastic litter present across coastal regions is derived from marine environments, transported and deposited through wave action (Browne et al., 2011). The suspension of plastic by aeolian processes is responsible for transferring particles from marine to atmospheric systems, with microplastics potentially aerosolized alongside the sea surface microlayer (Wright & Kelly, 2017). Plastic particles will also settle through the water column and become incorporated in marine sediments (Van Cauwenberghe et al., 2013). The rate at which this process occurs is influenced by amalgamation within faecal pellets (Cole et al., 2016; Porter, Lyons, Galloway, & Lewis, 2018) or incorporation into algal structures (Long et al., 2015). The accumulation of plastic in benthic sediments provides a temporary store which may be remobilized by physical and biological processes, although there is limited research on such mechanisms of plastic transport in marine systems (Martin, Lusher, Thompson, & Morley, 2017).

### 2.5 Under‐represented ecosystems

There are several ecosystems where the occurrence of plastics remains largely unexplored. In particular, groundwater and cryospheric ecosystems, as well as riparian ecotones, have received relatively limited attention. Yet the potential for these ecosystems to significantly influence the storage and flux of plastics could be substantial.

Within the cryosphere, the remobilization of plastics resulting from increasing melt rates may provide a significant source of plastics to other ecosystems. Existing research demonstrates high concentrations of plastic debris (40–250 MP/L melted ice) stored in Arctic sea ice (Obbard et al., 2014; Peeken et al., 2018). The release of plastic from sea ice is likely an important contributor to the flux of plastic within marine systems. As an example, the net melting of sea ice between 2011 and 2016 is estimated to have released 7.2–8.7 × 10^20 ^MP in the size range of 0.011–5 mm (Peeken et al., 2018). Within glaciated hydrological catchments, patterns of continuing deglaciation may lead to a significant release of plastic; however, little is known about the distribution of plastic contamination across these compartments of the cryosphere.

Groundwater systems provide important stores and transfer pathways of pollutants, for example, pesticides (Toccalino, Gilliom, Lindsey, & Rupert, 2014), so it is likely that these systems would store and transport micro‐ and nanoplastics (Rochman, 2018). While interstitial pore space within rock strata, hydrologic connectivity and subsurface flow paths limits potential plastic particle sizes, it is likely that some systems like karsts may also transport or store larger particle sizes. The relative contribution of groundwater to the total flux of plastic pollution, however, is likely restricted due to pore sizes.

Riparian ecotones, as the main interface between terrestrial and freshwater systems, are obvious locations for plastic transfer and storage. Recent studies have used citizen science techniques to quantify the levels of macroplastic litter along riverbanks and riparian zones, observing an average of 0.54 ± 1.2 litter items/m^2^ across Germany (Kiessling et al., 2019). Riparian zones likely provide temporally variable effects on the storage and transfer of plastic pollution. For example, during floods, plastics are prone to deposition above the bank, namely if the riparian vegetation increases retention. River level (water height), velocity, vegetation type, coverage and roughness are here key regulating factors in the storage, release or transport of plastics in riparian ecosystems. There, however, remains an absence of research surrounding the role of riparian zones in the transport of plastics across hydrological catchments.

## 3 BIOLOGICAL RETENTION AND CYCLING OF PLASTICS ACROSS CATCHMENTS

Plastics are transported, ingested, cycled and sometimes retained by biota. Biological interactions such as ingestion also alter the physical and chemical properties of these plastics, which in turn influences the movement (flux and storage) of plastic between ecosystems. As an example, as plastics are incorporated into faecal pellets, phytoplankton aggregates or biofilm matrices, the otherwise buoyant plastic particles gain a propensity to sink, leading to increased deposition in sediments (Cole et al., 2016; Long et al., 2015; Rummel, Jahnke, Gorokhova, Kühnel, & Schmitt‐Jansen, 2017). The aggregation of particles as a result of egestion may subsequently alter the distribution of plastics while also increasing their bioavailability to organisms feeding on faecal material (Ward & Kach, 2009). Once in food webs, plastic particles may be retained through transfers through multiple pathways (Windsor, Tilley, Tyler, & Ormerod, 2019) and cycling between trophic levels, moving upwards through the food web as a consequence of predation (e.g. Nelms, Galloway, Godley, Jarvis, & Lindeque, 2018) and re‐entering the basal resources through egestion. The residence time of plastic particles within the biological component of food webs is unknown. Higher plants may also retain plastic, with the potential for significant aerial accumulation, in the branches and foliage of plants in both terrestrial and riparian systems as well as entangled in subterranean and subaquatic plant material. The storage of plastics in the biotic components of ecosystems, ultimately however, is restricted with the majority of plastic particles likely to return to the environments from which they were sequestered, through a series of processes including egestion and decomposition (Wright, Thompson, & Galloway, 2013b).

Organisms may also facilitate the transport of plastics across habitats and ecosystems. For example, the dispersal of some organisms across the landscape may act to redistribute plastics at a range of spatial scales, from microhabitats to continents. Across short distances, organisms such as worms and collembolans may transport plastics via ingestion, attachment and active transport (Maaß, Daphi, Lehmann, & Rillig, 2017). Recent laboratory studies have also indicated the potential for mosquitoes (*Culex pipiens*; Linnaeus 1758), to transport microplastics (2 and 15 μm) from aquatic to terrestrial and atmospheric systems (Al‐Jaibachi, Cuthbert, & Callaghan, 2018). For microorganisms, transport may be relatively localized, yet larger organisms (e.g. cetaceans) may facilitate long distance transport. Such processes are likely responsible for distributing plastic over large distances thus generating plastic pollution in regions previously unaffected by nonbiological fluxes of plastics. These processes, however, are unlikely to be significant relative to redistribution by physical processes (e.g. winds and tides). The interaction between organisms and plastic transport, nevertheless, is an emergent field of research, requiring further attention.

## 4 ECOLOGICAL EFFECTS OF PLASTICS

**Figure 2:**
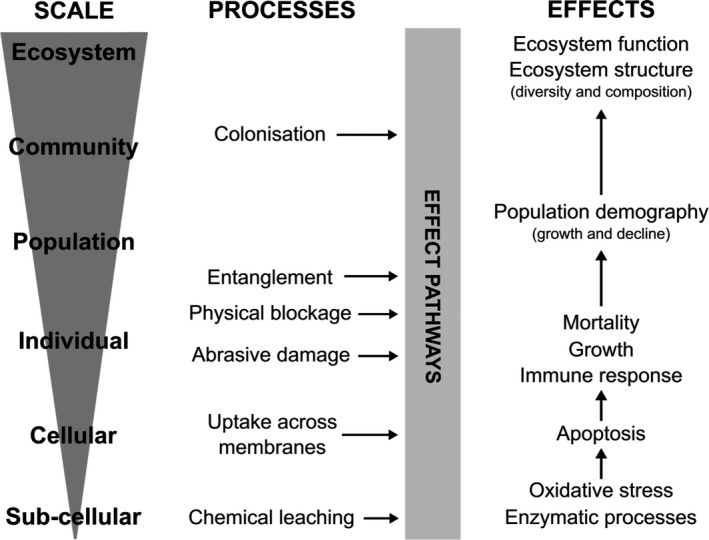
Observed and predicted mechanistic effects of plastic exposure in natural environments. Potential mechanistic effects are determined from theoretical and empirical studies, as well as perceived mechanisms of action which have yet to be investigated

Impacts on organisms and ecological processes from exposure to plastic may stem from an array of mechanisms. While current literature predominantly reports physical impacts on biota or ecosystem function, chemically related effects facilitated by the adsorption properties of plastic surfaces and the accumulation of hydrophobic chemicals, as well as the leaching of additives in particles, are also possible (Figure 2).

One of the largest bodies of observational evidence for the lethal effects of plastic pollution lies in records of entanglement and external physical damage. Although the majority of information available implicates large plastic items, for example, fishing nets and rope (e.g. Jacobsen, Massey, & Gulland, 2010), these physical effects also pose a problem for small organisms. For example, zooplankton exposed to microplastic fibres (1.7 × 10^4 ^– 5.4 × 10^5 ^fibres/L) were observed with antennal and carapace deformities resulting from external damage (Ziajahromi, Kumar, Neale, & Leusch, 2017). The concentrations utilized within this study, however, do not represent environmentally relevant concentrations. Observations in terrestrial systems have also identified the lethal effects of entanglement on American crow (*Corvus brachyrhynchos*; Brehm, 1822) nestlings (Townsend & Barker, 2014). The effects of entanglement, however, occur at the individual level, and there remains limited evidence to suggest that these frequently lethal impacts scale‐up to affect populations. Furthermore, the effects of plastic exposure on sensitive tissues have generally been carried out at concentrations exceeding those observed within natural environments (Phuong et al., 2016).

The ingestion of plastic has also been a focus of existing research with the severe effects (e.g. reduced growth and mortality) of plastic blockages in the digestive tracts of organisms attracting attention (Derraik, 2002; Gall & Thompson, 2015). These effects are observed across the biosphere, although they have so far been infrequently recorded on a small number of individuals. A range of more subtle effects, however, may be generated by plastic ingestion. The ingestion of plastic maintains the potential to generate reductions in the adsorption of nutrients by the organism (based on reduced uptake of nutrients and intake of actual food items), alterations in the gut microbiota and also reduce the energy budget of organisms leading to several subsequent impacts, including reduced feeding, decreased activity, reduced reproductive output and eventually mortality (see Wright, Rowe et al., 2013a; Au, Bruce, Bridges, & Klaine, 2015; Watts, Urbina, Corr, Lewis, & Galloway, 2015; Zhu et al., 2018). Thus far, exposure to a range of plastic types, sizes and shapes has generated relatively limited adverse effects on aquatic organisms, including fish and invertebrates (Foley, Feiner, Malinich, & Höök, 2018). As a specific example, a battery of six freshwater invertebrates exhibited limited responses in growth, reproduction and survival to polystyrene microplastics (20–500 μm) at concentrations of 0%–40% sediment dry weight (Redondo‐Hasselerharm, Falahudin, Peeters, & Koelmans, 2018). However, the complexity of plastics makes effects difficult to predict as the shape, size and type of polymer can influence particle toxicity. For example, microfibres have been shown to have a greater adverse effect than microbeads due to entanglement and carapace damage in water fleas (*Ceriodaphnia dubia*; Richard, 1894) (Ziajahromi et al., 2017).

In addition to physical effects, plastics can also leach toxic compounds (either additives within the plastic or environmental contaminants adsorbed to their surface), generating effects within organisms that come into contact with plastics. Plastics are complex compounds with a variety of added chemicals (plasticisers, hardeners, flame retardants, surfactants and synthetic dyes) to give them their specific properties. Over time, these additives leach out and can often act as toxic or endocrine disrupting chemicals within the environment (Hermabessiere et al., 2017). A wide range of toxic compounds have been identified as plastic additives, including bisphenol a (BPA), nonylphenol, polybrominated flame retardants and phthalates (Hermabessiere et al., 2017). These leachates have been shown to negatively affect development in the early life stages of invertebrates (Nobre et al., 2015), while also generating reproductive abnormalities in a range of organisms (Browne, Galloway, & Thompson, 2007).

Plastics may act as vectors within the environment, enhancing the transport of persistent organic pollutants (POPs) and other chemicals through biotic and abiotic components of ecosystems (Ziccardi, Edgington, Hentz, Kulacki, & Kane Driscoll, 2016). The “vector effect” has predominantly been portrayed as detrimental, with a range of harmful substances adsorbed to the surfaces of plastics (Koelmans, Bakir, Burton, & Janssen, 2016) and the possibility to potentiate the toxicity of other chemicals, for example, triclosan (Syberg et al., 2017). The role of microplastics in organic chemical bioaccumulation, however, is unclear. While previous studies have shown increased bioaccumulation of chemicals when adsorbed to plastics (Bakir, Rowland, & Thompson, 2014a; 2014b), recent evidence suggests that the role of microplastics in chemical transfer to organisms may be negligible when compared to other natural organic matter (Koelmans et al., 2016). Further to this, only a small fraction of contaminants appear to adsorb to the surface of common microplastics (polyethylene and polypropylene), with only hydrophobic compounds shown to consistently absorb to particles (Seidensticker, Grathwohl, Lamprecht, & Zarfl, 2018). Other studies have indicated that the presence of plastics during contaminant exposure maintains variable effects. For example, polystyrene microplastics (0.4–1.33 mm) provided a “cleaning” mechanism, whereby pollutants, in this case PCBs, are transferred from the tissues of the organisms to the microplastic particles (Koelmans, Besseling, Wegner, & Foekema, 2013). In another study, the addition of polyamide microplastic particles (15–20 μm) to experimental chambers reduced the aqueous concentrations of BPA, leading to a reduction in the levels immobilization of *Daphnia magna *(Straus, 1820) in comparison to exposure to only BPA (Rehse et al., 2018). The degree to which chemicals sorb to plastics is also highly variable and dependent on the environmental conditions (e.g. salinity, temperature, pH and organic matter), chemical characteristics and plastic type (Teuten et al., 2009). Although other substrates may provide a greater influence on the bioaccumulation of pollutants, the sorption of pollutants to plastics may enable the transfer of pollutants over greater distances compared to organic pollutants associated with denser sediment particles (Nizzetto, Bussi et al., 2016).

The surface of plastics provides a suitable substrate for colonization by microbial and invertebrate communities (McCormick et al., 2016; Reisser et al., 2014). Within urban river systems, plastics have been identified as a unique and important substrate for the colonization of aquatic microbial biofilms (McCormick, Hoellein, Mason, Schluep, & Kelly, 2014). Similar findings have been presented within marine systems, with diatoms, phytoplankton and cyanobacteria colonizing plastic particles suspended within the water column (Oberbeckmann, Osborn, & Duhaime, 2016; Reisser et al., 2014; Zettler, Mincer, & Amaral‐Zettler, 2013). While in some instances, the microbial communities on these plastic particles maintained comparable species richness and evenness to communities present on natural substrates (Zettler et al., 2013), other studies (e.g. McCormick et al., 2014) demonstrated that microbial communities inhabiting microplastic particles maintained a different taxonomic structure to those present in the water column and on suspended organic matter. An increasing body of research has also identified the colonization of plastic particles by harmful microbes, which could lead to further deleterious effect upon organisms interacting with these particles (Keswani, Oliver, Gutierrez, & Quilliam, 2016). For example, the ingestion of these particles may expose organisms to a range of adverse effects derived from harmful microbes and lead to long‐range transport of these microbes to regions that would not normally be found (Kirstein et al., 2016; Viršek, Lovšin, Koren, Kržan, & Peterlin, 2017). Further to this, recent studies have indicated that the intense interactions within microbial communities on microplastic particles enable the increased plasmid transfer between phylogenetically diverse bacteria, potentially facilitating the spread of antibiotic resistance across aquatic systems (Arias‐Andres, Klümper, Rojas‐Jimenez, & Grossart, 2018).

While individual‐level effects are widely demonstrated for macroplastics and in some cases microplastics, evidence for population and food web level effects remains restricted. As highlighted by Koelmans et al. (2017), a range of issues currently limit our understanding of the ecological risks resulting from exposure to plastic pollution. The majority of current individual‐level assessments suffer from three dominant limitations; (a) the absence of ecologically relevant metrics; (b) a limited understanding of organism‐plastic encounter rates for given exposure concentrations; and (c) the restricted development of dose–response relationships across suitable concentration ranges. As a result, the individual‐level and in some cases population effects identified within contemporary experimental assessments are not directly applicable to natural systems. Developing an improved mechanistic understanding of the effects of plastic pollution as well as following lessons learnt in previous environmental toxicology assessments (e.g. nonmonotonic relationships, mixture effects, indirect effects) is likely to improve our understanding of the ecological risks posed by plastic pollution.

## 5 UNDERSTANDING PLASTIC–BIOTA LINKS

The mechanisms through which plastic exposure effects occur are strongly dependent on the characteristics of plastic particles, including size, shape, colour and polymer type (Lambert, Scherer, & Wagner, 2017). As an example, polyvinyl chloride is generally more toxic than polyethylene and polypropylene, due to the greater toxicity of its additives and subsequent leachates (Lithner, Nordensvan, & Dave, 2012). The diversity of physical and chemical characteristics exhibited by plastic particles, throughout their lifecycle and as they degrade in natural systems, means that the potential ecological effects resulting from plastic pollution are extremely variable.

**Figure 3:**
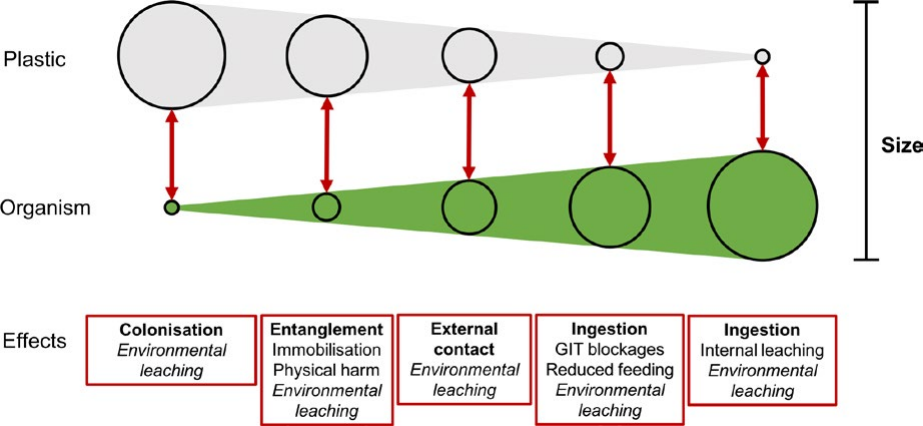
Simplified conceptual relationship between the organism‐to‐plastic size ratio and the dominant effects derived from direct interactions between organisms and plastic pollution at these scales. These relationships are independent of measured size, yet bounded by the maximum size of plastic particles and organisms in natural environments. Examples of potential effects at different size ratios are presented in red boxes. Bold text indicates the nature of organism‐plastic interactions and italic text indicates indirect effects

The relationship between organisms and plastic size appears particularly important in determining the nature and severity of ecological effects (Figure 3). Plastics significantly larger than the target organism can provide a novel substrate for colonization for the smaller organisms (as described for microbial communities (Reisser et al., 2014) and invertebrates (Davidson, 2012), or become a cause for entanglement and associated effects for larger organisms (Gall & Thompson, 2015). Plastics of large yet ingestible size classes present the potential for gastrointestinal blockages (Gall & Thompson, 2015). Finally, particles that are ingestible in size, yet too small to present physical risks (e.g. digestive blockages and entanglement), propose a large range of potential effects, including the leaching of toxic chemicals directly to organisms (e.g. Teuten et al., 2009). These general rules provide a good indication of the potential effects of different plastic particles; however, it should be noted that organisms are able to interact with all sizes of plastic pollution, with wide range of possible effects not detailed above. Further to this, the bioaccumulation and trophic transfer of particles make a wider range of plastics bioavailable to organisms that may not encounter particles or may experience higher concentrations than present in the environment (Carbery, O'Connor, & Palanisami, 2018; Nelms et al., 2018). A range of alternative indirect effects is also presented by particles of various sizes (Figure 3). As an example, chemicals from macroplastics leach into the surrounding environment, providing the potential to indirectly affect organisms through the uptake and subsequent effects.

Thus far, the observed effects of plastic pollution are mainly limited to the size classes utilized in experimental manipulations (0.04–500 μm) (Foley et al., 2018) or the size classes observed in fatalities in natural systems (0.3–10 m) (Jacobsen et al., 2010). Thus, the nature, mechanisms and severity of effects across the spectrum of plastic sizes are unknown. Further research investigating the interactions between organism size, plastic characteristics and ecological effects is important for developing a comprehensive knowledge of ecological risks posed by plastic pollution.

## 6 PLASTIC POLLUTION IN A SOCIAL AND ECONOMIC CONTEXT

Plastic has many societal benefits and has promoted a range of technological advances. However, increasing awareness of potential environmental impacts, hitherto focused predominantly on marine systems (Thompson, 2017), is also highlighting potential knock‐on effects across a range of economic sectors, including the water industry, tourism and fishing. Data are geographically restricted, yet indicate the potential for widespread socio‐economic effects of plastic pollution.

Fishing activity (commercial and recreational), in particular, is negatively impacted by plastic debris, reducing and damaging catches (Thompson, 2017); for example, 86% of Scottish fishing vessels surveyed had reported restricted catches as a result of marine litter (Mouat, Lopez‐Lozano, & Bateson, 2010). Furthermore, entanglement within marinas and harbours appears a significant problem, with 70% of surveyed marinas and harbours reporting that leisure users had experienced incidents with litter (Mouat et al., 2010). Contamination of fish stocks may also provide a significant economic cost, although the concentrations of plastic within individual fish are relatively low (e.g. 1–2 pieces per organism: Foekema et al., 2013; Lusher et al., 2013). Nevertheless, the negative perception of this contamination by consumers may be enough to affect the marketability of commercial organisms (GESAMP, 2016).

Another economic sector significantly impacted by plastic pollution is tourism. Public perceptions of plastic pollution are likely to influence where people choose to visit. For example, visitors to coastal regions cited the presence of litter as a factor influencing the locations they visited (Brouwer, Hadzhiyska, Ioakeimidis, & Ouderdorp, 2017). To mitigate the negative effects of litter, local authorities implement cleaning operations (Mouat et al., 2010). The combination of removal costs and potential reductions in tourism presents a major concern the tourism industry.

Expenses are also incurred through increased research and development relating to water treatment methods, damages to equipment and blockages of infrastructure. In particular, cosmetic wipes have been shown to cause problems – blocking sewage infrastructure and generating private and public effects (Drinkwater & Moy, 2017). The net costs of plastics to the water industry are, however, difficult to calculate as removal and blockages occur alongside other problematic items (e.g. fat, grease and organic pollutants).

Human health is potentially impacted by plastic pollution. Beach litter has been shown to cause physical harm (Werner et al., 2016); nevertheless, the vast majority of these incidents relate to metal and glass as opposed to plastic. Psychological effects of plastic litter are also observed with negative effects on the “restorative value” generated by visiting a polluted habitat (Wyles, Pahl, Thomas, & Thompson, 2016). The health of individuals may also be affected by any of the suite of effects highlighted in the previous section *Ecological effects of plastic*. This includes the transport of potentially harmful microbes and chemicals (see Keswani et al., 2016) as well as the physical effects of plastic ingestion. More work is nevertheless required to detail the specific health risks to human populations generated by global plastic pollution.

## 7 PLASTIC POLLUTION AS AN AGENT OF GLOBAL CHANGE

The relative impact of plastic pollution on ecosystems in comparison to other global stressors is poorly understood. Contextualizing the effects of plastic pollution within a multistressor environment is an important development, and to date, the importance of plastic effects in comparison to urbanization, habitat fragmentation, other pollutants, increased temperatures, hydrological changes and invasive species, for example, is unknown. Within the terrestrial environment, nevertheless, recent investigations across soil ecosystems, plastics have been identified as a potential agent of global change, altering the function of soils (water retention, microbial activity, soil structure and bulk density) and affecting their role in the function of the wider environment (de Souza Machado, Lau et al., 2018). Furthermore, microplastics have been shown to potentiate the effects of other xenobiotic pollutants, in this case the antimicrobial chemical triclosan (Syberg et al., 2017). The interactions between other stressors and plastic pollution therefore provide the potential to generate negative effects across natural ecosystems. Future mitigation and management strategies will require a better understanding of the relative importance of global pressures and also their interactions.

## 8 FUTURE RESEARCH AT THE CATCHMENT‐SCALE

Understanding the movement of plastic through hydrological catchments is an important step in determining the source to sink dynamics of plastics within natural systems. This review highlights that catchment‐scale assessments are currently mostly theoretical, but provide a framework to structure future investigations based on hypotheses generated by theoretical models. Supporting existing studies with comprehensive field‐based and experimental data sets is the logical next step in developing a comprehensive body of research assessing catchment‐scale transport and effects of plastic pollution. To date, empirical studies have focused on individual ecosystems providing an analysis of plastic distribution and plastic–organism interactions. Catchment‐scale assessments are an important next step for research, particularly to underpin the management of plastic sources from a more informed perspective. Several important developments required to facilitate the advance of catchment‐scale investigations are detailed in the following sections.

### 8.1 Methods for tracing plastic transport processes

Contemporary empirical assessments are not able to elucidate the sources and pathways of plastic particles, as once particles enter the environment, tracing sources becomes problematic. Furthermore, the longer particles are exposed to physical, chemical or biological processes, the more their transformation exacerbates difficulties identifying sources. Novel methods of tracing plastics have yet to be developed, yet using tracer studies to support existing models will allow for directed research projects attempting to bridge current knowledge gaps.

### 8.2 Hotspots and sinks of plastic pollution

Knowledge surrounding the distribution of plastic pollution across catchments is limited. Understanding where and how high plastic concentrations arise in space and time is required for assessments detailing how plastic concentrations may vary across hydrological catchments. The importance of such developments is further emphasized by a recent study which identified the highest concentration of microplastics yet recorded within riverine sediments globally (517,000 MP/m^2^) (Hurley et al., 2018). Assessments of heterogeneity are required at a range of spatial scales, from local patch dynamics at centimetre to metre scales, to comparisons between entire habitats and ecosystems. Understanding spatial variation and potential sinks of plastic will allow for an improved understanding of transport processes leading to the deposition of plastics across the landscape and importantly provide more accurate risk maps for biota.

### 8.3 Quantification of source contributions

Although estimates exist for the net contribution of plastic from specific ecosystems, for example, freshwater (Lebreton et al., 2017) and terrestrial (Horton et al., Horton, Svendsen et al., 2017) systems, the importance of specific sources in contributing to these plastic burdens across these environments is poorly understood. Further study of plastic sources, in particular diffuse contributions, is required to better resolve the source–flux–sink nexus within catchments, detailed in previous sections. Developing more accurate methods of quantification designed to detect low concentrations of plastic and nanoplastics will enable the detection of a wider range of plastics (e.g. tyre dust), allow for an improved understanding of plastic pollution across catchments and bridge the current gap between estimated inputs of plastic into catchments and measured environmental concentrations. Furthermore, standardizing measurements across samples to allow for comparison among studies, sources and environment is important (Filella, 2015), with the diversity of current measurements limiting an understanding of the relative concentrations of plastic pollution across the environment. Through investigating the characteristics and concentration of plastics released from each potential source, a mixing‐model type assessment can be used to understand the entrance and flux of plastics within catchments (Fahrenfeld, Arbuckle‐Keil, Naderi Beni, & Bartelt‐Hunt, 2018). Further to this, determining the specific contributions from sources will enable targeted mitigation, ultimately aimed at preventing the entrance of plastics into the natural environment.

### 8.4 Determining the applicability of catchment assessments

Catchment‐scale assessments are dependent on catchment characteristics, including but not limited to size, relief, land cover, water quality, hydrological connectivity and geomorphological features. The degree to which plastic studies within individual catchments are applicable across the wider landscape is unknown. To answer this question, multiple catchment assessments are required to determine the relative importance of catchment‐specific processes (e.g. hydrological flow paths, subsurface characteristics and catchment geology) in comparison to more generalizable characteristics (e.g. land cover, population density, human activities). An understanding of the importance of processes at a range of spatial and temporal scales is also required in order to appreciate the extent to which relationships are applicable across catchments.

### 8.5 Progressing from descriptions of the occurrence of plastics within catchments to assessing ecological effects

Given the increasing number of studies detecting or illustrating the ubiquity of plastics in global ecosystems, including across catchments, we suggest a need for a move to understanding effects on populations, communities and ecosystem functions, for example, food web transfer.

## 9 CONCLUSIONS

Our understanding of the effects of plastics within ecosystems indicates the potential negative effects of these pollutants when present in smaller fragments as well as macrofragments. Knowledge regarding the nature and severity of effects derived from smaller plastic particles, at environmentally relevant concentrations, however, remains restricted. The array of mechanistic effects identified by studies nevertheless indicates the potential for adverse effects within natural systems. The significant potential for effects coupled with recent research indicating the relative global ubiquity of plastics provides a perceivable risk to a range of ecosystems. In spite of this, we are only starting to understand the fluxes and pools of plastics within a range of ecosystems. This knowledge is nonetheless fundamental for mitigating existing and future plastic pollution. It is apparent that further research is required to better understand the interactions between plastic pollution and organisms in many ecosystems. Furthermore, a comprehensive understanding of potential ecological risks presented by plastics remains absent with a range of potential adverse effects remaining unexplored. The existing ecological risk presented by plastic pollution is estimated to continue into the future as a result of predicted increases in production of plastics, the significant persistence of plastic particles and the degradation of existing plastic pollution generating increases in micro‐ and nanoplastic concentrations across the globe.

## CONFLICTS OF INTEREST

The authors declare no conflicts of interest.
